# Does aneurysm side influence the infarction side and patients´ outcome after subarachnoid hemorrhage?

**DOI:** 10.1371/journal.pone.0224013

**Published:** 2019-11-07

**Authors:** Nina Brawanski, Sepide Kashefiolasl, Sae-Yeon Won, Stephanie Tritt, Joachim Berkefeld, Christian Senft, Volker Seifert, Jürgen Konczalla

**Affiliations:** 1 Department of Neurosurgery, Goethe-University Hospital, Frankfurt am Main, Germany; 2 Institute of Neuroradiology, Goethe- University Hospital, Frankfurt am Main, Germany; 3 Institute of Neuroradiology, Helios HSK Wiesbaden, Wiesbaden, Germany; Universitatsklinikum Freiburg, GERMANY

## Abstract

**Background:**

The prognostic factors and outcome of aneurysms appear to be dependent on its locations. Therefore, we compared left- and right- sided aneurysms in patients with aneurysmal subarachnoid hemorrhage (SAH) in terms of differences in outcome and prognostic factors.

**Methods:**

Patients with SAH were entered into a prospectively collected database. A total of 509 patients with aneurysmal subarachnoid hemorrhage were retrospectively selected and stratified in two groups depending on side of ruptured aneurysm (right n = 284 vs. left n = 225). Midline aneurysms of the basilar and anterior communicating arteries were excluded from the analysis. Outcomes were assessed using the modified Rankin Scale (mRS; favorable (mRS 0–2) vs. unfavorable (mRS 3–6)) six months after SAH.

**Results:**

We did not identify any differences in outcome depending on left- and right-sided ruptured aneurysms. In both groups, the significant negative predictive factors included clinical admission status (WFNS IV+V), Fisher 3- bleeding pattern in CT, the occurrence of delayed cerebral ischemia (DCI), early hydrocephalus and later shunt-dependence. The side of the ruptured aneurysm does not seem to influence patients´ outcome. Interestingly, the aneurysm side predicts the side of infarction, with a significant influence on patients´ outcome in case of left-sided infarctions. In addition, the in multivariate analysis side of aneurysm was an independent predictor for the side of cerebral infarctions.

**Conclusion:**

The side of the ruptured aneurysms (right or left) did not influence patients’ outcome. However, the aneurysm-side predicts the side of delayed infarctions and outcome appear to be worse in patients with left-sided infarctions.

## Introduction

Recently published data have shown differences in prognostic factors and outcome depending on aneurysm location[1,2]. Additionally, factors including an existing concomitant intracerebral hemorrhage or hydrocephalus are known as factors influencing patients´ outcome. Furthermore, studies have reported different results concerning strokes that occur in the dominant or non- dominant hemisphere in terms of patients´ outcome. On the one hand Sposato et al[3] found an association between right insular cortex ischemic stroke and increased mortality and poor functional outcome at six months. On the other hand in stroke with infarctions in the dominant hemisphere Sundseth et al. showed no statistical or clinical difference in functional outcome and quality of life in patients with speech-dominant stroke compared to non-dominant side infarction[4].

In this study, we compared all left- and right-sided aneurysms in patients with aneurysmal subarachnoid hemorrhage (SAH) in our department in terms of differences in outcome and prognostic factors. We also aimed to determine whether infarctions in the dominant hemisphere can significantly influence patients´ outcome.

### Methods

This study was approved by the local ethics committee of the Goethe- University Hospital Frankfurt.

### Study population

Patients, who were admitted to our hospital with a diagnosis of SAH were selected and collected in a prospectively conducted database (IBM SPSS ^®^, Armonk, NY, USA). Patients with traumatic, non- aneurysmal SAH and not- treated patients were excluded. We included aneurysms of A1, M1-M4, posterior inferior cerebellar artery (PICA), posterior cerebral artery (ACP), posterior communicating artery (ACoP) and internal carotid artery (ICA), as well as aneurysms at a distance of over one cm from the midline. Basilar artery aneurysms and aneurysms of anterior communicating artery were excluded from analysis. All patients who underwent a ´crossover´- procedure were stratified by the final occlusion (e.g. first attempt coiling and finally microsurgically treatment were rated to the clipping cohort). In total, 509 patients were selected and divided in two groups depending on the side of ruptured aneurysm (right n = 284 vs. left n = 225).

All patients were admitted to our neuro-intensive care unit (ICU). An existing early hydrocephalus was defined as the need for attachment of an external ventricular drain. In our institution all patients with proven hydrocephalus (extension of internal ventricular system, especially in comatose patients or those with reduced vigilance) received an external ventricular drain (EVD) before any further diagnostic or therapeutic procedure. We do not place a lumbar drain or EVD prior to surgery to achieve brain relaxation. Patients regularly received a digital subtraction angiography (DSA) and after interdisciplinary consensus aneurysm treatment (by clip or coil) [5,6]. All patients received nimodipine admission (60mg six times/ 24hours) until day 21.

Neurological observation and transcranial duplex (TCD) were performed at least once a day. All patients received routine a cranial imaging the first day after aneurysm treatment. On day 7 patients routinely underwent imaging studies including angiography (DSA after microsurgical clipping or MR/CT-angiography after endovascular coiling). When there was suspicion of cerebral vasospasm (CVS) (neurological deterioration or increased TCD values) further diagnostic imaging via computed tomographic angiography (CTA) or magnetic resonance angiography (MRA) was performed. CVS was defined as a visible reduction in diameter in cranial imaging in comparison to initial imaging. Vessel reduction in cranial imaging was classified into four grades: No CVS (no vessel reduction in comparison to initial imaging), mild CVS (vessel reduction up to 33%), moderate CSV (vessel reduction from 34–66%) and severe CVS (reduction in vessel diameter over 66%). In awake patients and in unconscious patients with the possibility of neurological assessment (NIHSS and Glasgow coma scale (GCS)) neurological deterioration after exclusion of other causes and at least moderate to severe CVS (33–66% or >66% respectively) were defined as delayed cerebral ischemia (DCI) and were included in the statistical analysis[7,8]. In unconscious patients without the possibility of neurological assessment (deep sedation with a GCS score of 3) only patients with vessel reduction >66% (severe CVS) were included for DCI-analyses. For DCI/vasospasm treatment we induced hypertension aiming for a mean arterial blood pressure over 100 mmHg. Endovascular therapy (application of intra-arterial nimodipine and/ or angioplasty) also was applied in selected patients.

Due to incomplete recording of the dominant hemisphere in selected patients at the time of admission (right- or left- handedness), this information could not be consistently collected and therefore could not be used for analysis.

All cranial imaging was examined by an experienced neuroradiologist (ST), who differentiated between delayed infarctions and infarctions which most likely had a procedural- related root cause. Most infarctions were confirmed by magnetic resonance imaging (MRI). Patients with procedure- related infarctions or infarctions due to herniation were not included in the analysis.

Patient outcome was assessed using the modified Rankin Scale (mRS) six months after SAH (favorable outcome mRS 0–2 vs. unfavorable outcome mRS >2). Assessment of mRS was performed by trained neurosurgeons. The outcome was assessed during a regular outpatient visit or via a structured telephone call.

### Statistical analysis

Statistically analyses were performed using commercially available software (IBM SPSS ^®^Inc. 22, Armonk, NY, USA). Categorical variables were analyzed using the Fisher exact test and unpaired t- test with Welch correlation for parametric values. Probability values <0.05 were considered statistically significant. A multivariate analysis was subsequently performed to identify independent predictors of a favorable outcome after six months and to identify any confounding effects between potentially independent predictors. Variables from the univariate analyses were considered to be potentially independent factors in the multivariate analysis. A backward stepwise method was used to construct a multivariate logistic regression model considering favorable outcome as a dependent variable with an inclusion criterion of p < 0.1.

## Results

### Patients´ characteristics

In total 509 patients with a mean age of 54.1 ± 13.9 years were included in the study. Of these patients 225 (44.2%) suffered from an aneurysm of the left side and 284 (55.8%) from the right side.

On admission, 276 patients (52.5%) had a good clinical status (measured by World Federation of Neurological Surgeons-(WFNS)- grade I- III). A total of 78 patients (15.3%) died. Early hydrocephalus was detected in 309 patients (60.7%). 100 patients (19.6%) required a ventricular-peritoneal (VP) shunt. Detailed data are presented in Table 1.

**Table 1:** Patient characteristics.

Patient characteristics	All patients	Right-sided aneurysms	Left-sided aneurysms	P value (right vs. left)	OR (95% Cl)
**Number of patients**	509	284 (55.8%)	225 (44.2%)		
**Mean age (years)**	54.1 ± 13.9	54.2 ± 13.1	54.1 ± 14.9	NS (0.9)	
**Female sex**	376 (73.9%)	212 (74.6%)	164 (72.9%)	NS (0.7)	
**Worse admission status (WFNS ≥4)**	242 (47.5%)	126 (44.4%)	116 (51.6%)	NS (0.1)	
**Early hydrocephalus**	309 (60.7%)	162 (57%)	147 (65.3%)	NS (0.08)	
**Shunt dependence**	100 (19.6%)	53 (18.7%)	47 (20.9%)	NS (0.8)	
**Fisher grade 3**	368 (72.3%)	202 (71.1%)	166 (73.8%)	NS (0.5)	
**Coil**	231 (45.4%)	132 (46.5%)	99 (44%)	NS (0.6)	
**DCI**	101 (19.8%)	56 (19.7%)	45 (20%)	NS (1)	
**Right-sided infarctions**	83 (16.3%)	76 (26.8%)	7 (3.1%)	< 0.0001	0.1 (0.04–0.2)
**Left-sided infarctions**	70 (13.8%)	11 (3.9%)	59 (26.2%)	< 0.0001	8.8 (4.5–17.3)
**Favorable outcome** **(mRS 0–2)**	300 (58.9%)	175 (61.6%)	125 (55.6%)	NS (0.2)	
**Unfavorable outcome** **(****mRS 3–6****)**	209 (41.1%)	109 (38.4%)	100 (44.4%)	NS (0.2)	
**Death (mRS 6)**	78 (15.3%)	41 (14.4%)	37 (16.4%)	NS (0.5)	

### Outcome characteristics

A total of 300 patients (58.9%) showed a favorable outcome (mRS 0–2) six months after SAH. In univariate analysis the admission status, early hydrocephalus, Fisher 3 bleeding pattern, severe cerebral vasospasm and shunt dependence were found to have a significant influence on patients´ outcome (see Table 2). In addition, the occurrence of left- sided cerebral infarctions was significantly associated with an unfavorable outcome (p = 0.0002), whereas right- sided infarctions were not (p = 0.1) (see Table 2). Independent risk factors for an unfavorable outcome in the multivariate analysis were a poor admission status (WFNS ≥4), age over 60 years, DCI and patients with left- sided infarctions (details are shown in Table 2).

**Table 2:** Outcome characteristics in all patients (uni- and multivariate analysis).

				Univariate analysis		Multivariate analysis	
Patient characteristics	All patients	Favorable outcome mRS 0–2	Unfavorable outcome mRS 3–6	P value	OR (95% Cl)	P value	OR (95% Cl)
**Number of patients**	509	300 (58.9%)	209 (41.1%)				
**Mean age (years)**	54.1 ± 13.9	51.6 ± 12.3	57.8 ± 15.2	< 0.0001	6.2 (3.7–8.6)		
**Age 60+**	168 (33.0%)	73 (24.3%)	95 (45.5%)	< 0.0001	2.59 (1.77–3.79)	< 0.0001	2.6 (1.6–4.0)
**Female sex**	376 (73.9%)	227 (75.7%)	149 (71.3%)	NS		NS	
**Worse admission status (WFNS ≥4)**	242 (47.5%)	92 (30.7%)	150 (71.8%)	< 0.0001	5.0 (3.3–10)	< 0.0001	5.1 (3.1–8.4)
**Early hydrocephalus**	309 (60.7%)	150 (50%)	159 (76.1%)	< 0.0001	3.2 (2.2–4.7)	NS	
**Shunt dependence**	100 (19.6%)	42 (14%)	58 (27.8%)	< 0.0001	2.4 (1.5–3.7)	NS	
**Fisher grade 3**	368 (72.3%)	201 (67%)	167 (79.9%)	0.001	2.0 (1.3–3.0)	NS	
**Coil**	231 (45.4%)	142 (47.3%)	89 (42.6%)	NS		NS	
**DCI**	101 (19.8%)	40 (13.3%)	61 (29.2%)	< 0.0001	2.7 (1.7–4.2)	0.0005	2.3 (1.3–4.2)
**Left-sided infarctions**	70 (13.7%)	27 (9%)	43 (20.6%)	0.0002	2.6 (1.6–4.4)	0.002	2.3 (1.4–3.8)
**Right-sided infarctions**	83 (16.3%)	42 (14%)	41 (19.6%)	NS		NS	
**Left-sided aneurysms**	225 (44.2%)	125 (41.7%)	100 (47.8%)	NS		NS	
**Right-sided aneurysms**	284 (55.8%)	175 (58.3%)	109 (52.2%)	NS		NS	

### Prognostic factors depending on aneurysm side

For right- and left-sided aneurysms risk factors for an unfavorable outcome were a worse admission status (WFNS≥4), the occurrence of an early hydrocephalus (p< 0.0001), a later shunt dependence (p = 0.002) and the occurrence of DCI (p< 0.0001). For left sided-aneurysms Fisher grade 3 bleeding pattern was a significant predictor, but this finding did not reach statistical significance in for right-sided aneurysms. Additionally, the side of the ruptured aneurysm appeared to play an important role with regard to the infarction- side. In patients with left- sided aneurysms significantly more left- sided infarctions were detected, whereas patients with right- sided aneurysms were more prone to developing right- sided infarctions. Details are shown in S1 Table.

### Comparison of left- and right-sided ruptured aneurysms

**Fig 1:**
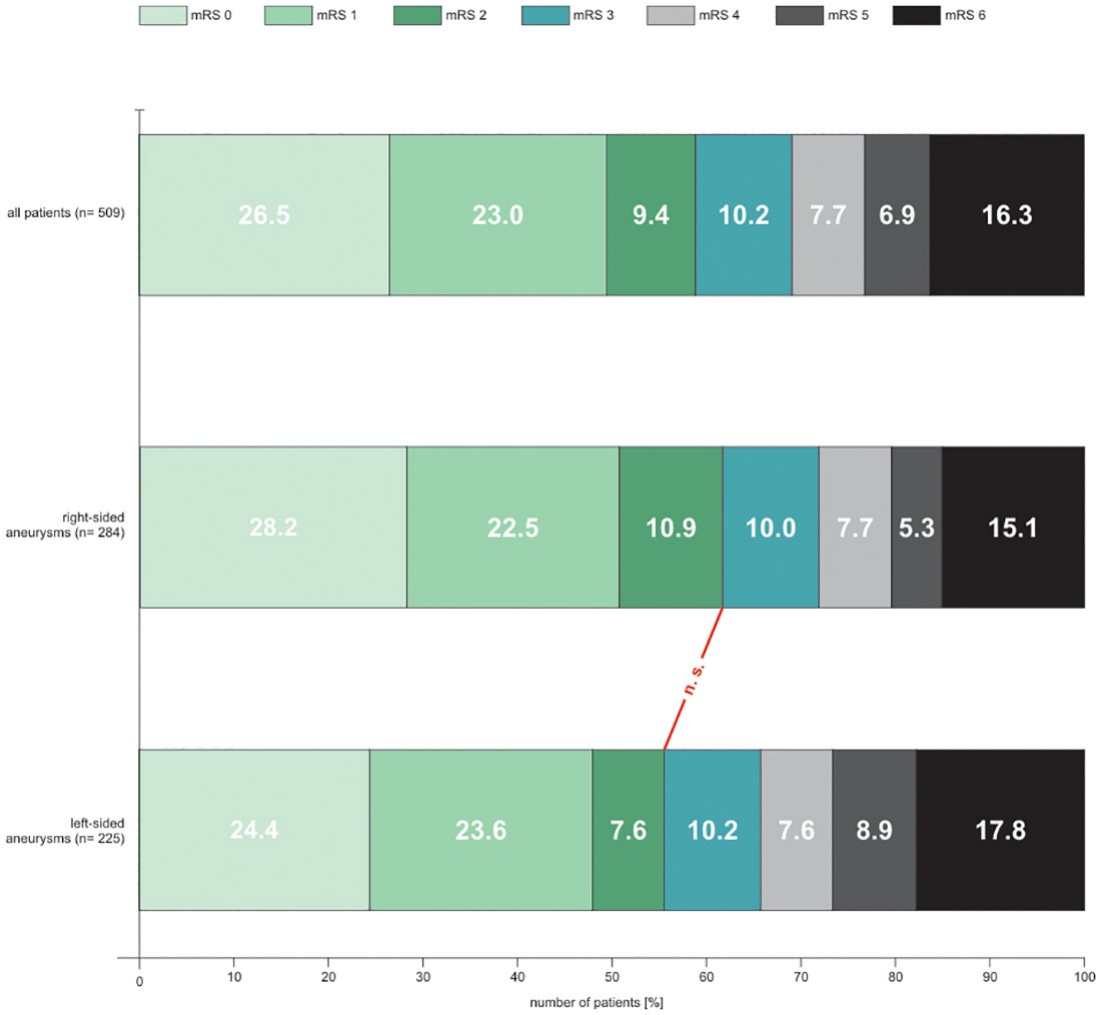
Outcome in right- and left- sided aneurysms.

When left- and right-sided ruptured aneurysms were compared, there were no significant statistically differences for the well- known prognostic factors such as the admission- status (WFNS), early hydrocephalus, shunt- dependence, Fisher-grade 3 bleeding pattern and DCI. Comparing outcomes in both groups, the statistical analysis showed no difference between the two groups of patients (details are shown in Fig 1). Furthermore, when the patient groups were compared of both patient groups statistical analysis showed a higher rate of infarctions on the corresponding aneurysm side. Detailed results are presented in S1 Table.

### Prognostic factors for left sided infarctions

As left-sided infarction was identified as an independent risk factor for an unfavorable outcome, we subsequently performed a multivariate analysis. Independent risk factors for left-sided infarctions were a worse admission status (WFNS ≥4), DCI and a ruptured left-sided aneurysm. In the statistical analysis, although older age was identified as an independent risk factor for right- sided infarctions, for left- sided infarctions there was only a trend (p = 0,3 OR 1.3 (0.8–2.0)), which was most likely due to the only small number of left- sided infarctions observed in our patients. In addition, the rate of infarctions was higher in elderly patients compared to younger patients (3 of 168 (37,5%) vs. younger 90 of 341 (26.4%); p = 0.01 OR 1.7 (1.1–2.5)). Detailed results are presented in Table 3.

**Table 3:** Independent risk factors influencing the occurrence of right- and left- sided infarctions (multivariate analysis).

**Independent risk factors for right- sided infarctions**		
**Risk factors**	**P value**	**OR (95% Cl)**
Worse admission status (WFNS ≥4)	< 0.0001	2.4 (1.5–4.0)
DCI	< 0.0001	4.6 (2.6–8.1)
Age ≥ 60 years	0.004	2.1 (1.3–3.5)
Right- sided aneurysm	< 0.001	7.0 (3.9–12.4)
**Independent risk factors for left- sided infarctions**		
**Risk factors**	**P value**	**OR (95% Cl)**
Worse admission status (WFNS ≥4)	< 0.0001	3.9 (2.4–6.4)
DCI	0.01	2.0 (1.2–3.4)
Left- sided aneurysm	< 0.0001	3.3 (2.0–5.4)

## Discussion

Cerebral infarctions after aneurysmal SAH are known as to be significant prognostic factors for patients´ outcome[9]. Abla et al. published data from 2014 regarding the relationship between aneurysm location and the incidence of CVS.[1] However, the authors did not look for association between the aneurysm side, the occurrence of infarctions and patients’ outcome. The exact impact of DCI on patients´ outcome remains unknown. For further analysis, a currently ongoing study by Stienen et al [9], will evaluate the relationship between DCI and outcome in affected patients in a prospective, observational, multicenter study (MoCA-DCI study), in which patients will be recruited until end of July 2019. Another study recently published showed that already early alterations in perfusion- computed tomography may identify patients with high risk of developing DCI[10] and so can improve patient management in the sense of early DCI detection and a following appropriate treatment.

To evaluate the prognostic factors after aneurysmal SAH, we compared right- and left-sided ruptured aneurysms. We questioned whether there are any differences in patient outcome and in the occurrence of cerebral infarctions according to the side of the ruptured aneurysm. So, Infarctions in the dominant hemisphere (left hemisphere in over 90% of the population may lead to worse outcome after aneurysmal SAH and can cause symptoms like speech-arrest and dysfunctions in writing and understanding causalities in spoken and written language.

### Outcome and prognostic factors

Statistical analysis showed no differences in the typical prognostic factors between the two examined patient groups, including a younger age, WFNS- grade at the time of admission, early hydrocephalus, a later shunt dependence and the occurrence of DCI. These factors are already recognized as prognostic factors that influence the outcome in patients after SAH[11,12]. Furthermore, a study published in 2018 showed, that the ´home- time´ for the first 90 days after SAH offers an easily applicable outcome measure for affected patients[13]. Zumofen et al [14] showed a correlation between chronological age, aneurysm location, aneurysm- size and pre- ictal functional status, and the influence on patients´ admission status and outcome. Other studies have described an influence on early infarctions and a therefore a worse outcome in affected patients[15]. However, studies providing detailed look information on the association between infarction and aneurysm location (which hemisphere) are scarce or missing[3,4].

### Comparison of right- and left- sided aneurysms

We identified that the side of infarction is correlated with aneurysm side. Furthermore, patients with left-sided aneurysms had significantly more frequent infarctions in the left (dominant) hemisphere.

Accordingly, in patients with ruptured right-sided aneurysm the right hemisphere was affected significantly more often. Comparing the outcome of both patient groups, while there was statistical difference, there was a trend toward a better outcome in patients with right- sided infarctions (61% favorable outcome in patients with right- sided infarctions vs. 55% favorable outcome in patients with ruptured left- sided aneurysm), which also can be explained by the possible presence of lesions in the dominant hemisphere caused by SAH[16].

We identified that aneurysm side predicted the hemisphere that would be affected by the delayed infarction. Although the Fisher 3 –bleeding pattern was not found to be a statistical significant prognostic factor in our study, the maximal blood thickness after SAH has been reported to be a predictor for DCI and an unfavorable outcome[17]. In our study only left-sided infarctions were a statistically significant negative prognostic factor for patients´ outcome, while right-sided infarction did not seem to influence patients’ outcome. This result is also consistent with to the trend toward a better outcome in ruptured right-sided aneurysm and accordingly a trend to a worse outcome in patients with left-sided ruptured aneurysms (favorable outcome in right- sided aneurysms of 61% vs. favorable outcome in left- sided aneurysms of 55%). In patients with an acute right- or left- hemispheric stroke a recurrent neglect syndrome, which is defined as a failure to orientate, report or respond to relevant stimuli on the opposite side to the lesion, is a predictor of a later unfavorable outcome in affected patients[18]. Moreover, in stroke- patients lesion location is known to be a significant predictor for patients´ outcome[19], as well as for the influence on infarct volume and the occurrence of multiple infarcts on outcome[20–22]. Other studies have described a higher rate of neglect phenomena and related disorders in right- hemispheric stroke[23–25]. On the other hand visual disorders or a visual neglect were found to be predominantly associated with left- sided stroke[18]. In contrast, other studies have described no differences in outcome between dominant and non- dominant cerebral lesions[26] and some have even reported a poorer outcome in patients who suffer a stroke in the non- dominant hemisphere (right hemisphere)[27,28]. In aneurysmal SAH there are no differences in relation to summative cognitive scores like the Mini Mental State Examination (MMSE) and Montreal Cognitive Assessment (MoCA) when comparing patients with left- and right- sided infarctions[29], but cortical infarctions in the middle cerebral artery region and perforator- infarctions were significantly associated with pathological changes in MMSE, MoCA and mRS[16].

While the outcome was not correlated with the side of the aneurysm, we observed an association with the side of the infarction. There was a trend toward a better outcome in patients with right- sided infarctions, which can be explained by the occurrence of the lesions in the non-dominant hemisphere [20].

Therefore, although the aneurysm side, did not influence the patients’ outcome per se, it was a strong predictor for side of infarction. Furthermore, an infarction in the left dominant hemisphere impairs patients´ outcome after aneurysmal SAH. Although all patients with aneurysmal SAH need best possible treatment to avoid complications especially in the prevention of cerebral vasospasm and infarctions, which can be most effective with a standardized treatment[10]. However, maybe our results can sensitize to be even more attentive in patients with left sided aneurysms and neurological deterioration, as we identify, that the occurrence of left sided infarctions will worsen outcome in these affected patients. In our opinion, it is helpful to know side- dependent differences in outcome and prognostic factors assist in the choice of treatment with the aim to prevent complications and improve patients´ outcome. This is particularly important for preventing left- sided infarctions, as patient care can be intensified in those patients identified as being ´high risk´ (patients with a statistically higher risk for left-sided infarctions). Additionally, having concrete numbers for favorable and unfavorable outcome, will assist in discussions with patients´ relatives, especially for seriously affected patients, in order to make the best therapy decision for these patients.

The independent risk factors for an unfavorable outcome identified by the multivariate analysis were a poor admission status (WFNS ≥4), age over 60 years, DCI and left- sided infarctions (details are shown in Table 2). In patients with cerebral stroke, lesion location is known as a significant predictor for patients´ outcome[12], as well as the influence on infarct volume and the occurrence of multiple infarcts on outcome[13, 15, 16] and worsen patients´ outcome. Another study confirmed that DCI and cerebral infarctions are independent factors for in- hospital mortality[30]. In this context a higher rate of neglect phenomena and most related disorders are described for right hemispheric stroke [5–7], while visual disorders or a visual neglect were mostly mainly associated with left- sided stroke[18].

Other stroke studies have found no differences in outcome between dominant and non- dominant cerebral lesions[4] or even a poorer outcome in patients with stroke in the non- dominant hemisphere (right hemisphere)[2, 11]. However, in the current study we identified a significantly poorer outcome in patients with left sided infarctions, whereas right sided infarctions did not significantly affect the outcome (Table 1).

We identified that the aneurysm side seems to predict side of delayed infarctions. These results were confirmed in the multivariate analysis, in which left- sided aneurysms were an independent predictor for left-sided infarctions and vice versa, which can possibly be explained by a higher rate of CVS in the region of ruptured aneurysms (especially on the side of the ruptured aneurysm) with a higher blood concentration in the area around the ruptured aneurysm and consequently higher rates of DCI/ CVS.

### Limitations and generalizability

The study has several limitations. Firstly, although this was a retrospective, single center statistical analysis, all data were collected prospectively. Because of the retrospective design, there are the typical limitations, such as data that were not documented initially in the medical records, like the dominant hemisphere. However, dominant hemisphere is located on the left side in over 90% of individuals, the patient cohort was relatively large and the multivariate analysis strengthens the results.

For clinicians it is important to know, that the aneurysm side and treatment strategy do not appear to influence the outcome. However, clinicians should be on alert, for vasospasm and clinical deterioration especially in patients with left-sided aneurysms, as they are associated with significantly more infarctions in the dominant hemisphere, which lead to a worse outcome. Additional data and afterwards a meta-analysis including a larger group of patients would be helpful to strengthen the power of our results.

## Conclusion

We identified that the aneurysm side, did not influence the patients’ outcome per se, but is a significant predictor for the side of infarction. Our results show a higher incidence of infarctions on the corresponding side of the ruptured aneurysm and prognostic relevance in the case of left-sided infarctions.

Although all patients with aneurysmal SAH should achieve a standardized treatment to avoid typical complications after SAH, left-sided infarctions had a more negative influence on patients´ outcome. This can be explained by the presence of lesions in the dominant left hemisphere, which can cause speech arrest and problems understanding spoken and written words. Therefore, cerebral infarctions in the left hemisphere should be avoided especially in patients with left- sided aneurysms due to the significant influence on the outcome of affected patients.

## Supporting information

S1 Table Prognostic factors in right- and left-sided aneurysms and comparison of both patient group. (DOCX)

S1 File Dataset. (XLS)

## Abbreviations

ACoP: posterior communicating artery
ACP: posterior cerebral artery
CBF: cerebral blood flow
CCT: cranial computed tomography
CTA: computed tomography angiography
CVS: cerebral vasospasm
DC: decompressive craniectomy
DCI: delayed cerebral ischemia
DSA: digital subtraction angiography
EVD: external ventricular drainage
GCS: Glasgow coma scale
ICA: internal carotid artery
ICH: intracerebral hematoma
ICU: Intensive care unit
MCA: middle cerebral artery
MMSE: Mini Mental State Examination
MoCA: Montreal Cognitive Assessment
MRA: magnetic resonance angiography
MRI: magnetic resonance imaging
mRS: modified Rankin Scale
n: number
OR: odds ratio
P values: probability values
PICA: posterior inferior cerebellar artery
SAH: subarachnoid hemorrhage
TCD: transcranial duplex
VP- shunt: ventricular- peritoneal shunt
vs.: versus
WFNS: World Federation of Neurological Surgeons
