# Supplementary material for: Does aneurysm side influence the infarction side and patients´ outcome after subarachnoid hemorrhage?
Source: PLoS One. 2019 Nov 7;14(11):e0224013. doi: 10.1371/journal.pone.0224013 (PMC6837438; doi:10.1371/journal.pone.0224013)
Supplement: S1 Table — (DOCX) [file pone.0224013.s001.docx]

**S1 table: Prognostic factors in right- and left-sided aneurysms and comparison of both patient groups**

| **Patient characteristics** | **Right-sided aneurysms** | **Favorable outcome**  **mRS 0-2** | **Unfavorable outcome**  **mRS 3-6** | **P value**  **OR (95% Cl)** | **Left-sided aneurysms** | **Favorable outcome**  **mRS 0-2** | **Unfavorable outcome**  **mRS ~~>2~~ 3-6** | **P value**  **OR (95% Cl)** | **Comparison**  **right- vs. left-sided aneurysms** |
| --- | --- | --- | --- | --- | --- | --- | --- | --- | --- |
| **Number of patients** | 284 (55.8%) | 175 (61.6%) | 109 (38.4%) |  | 225 (44.2%) | 125 (55.5%) | 100 (44.4%) |  |  |
| **Mean age (years)** | 54.2 ± 13.1 | 51.7 ± 11.8 | 58.2 ± 14.1 | 0.0001  6.5 (3.4- 9.6) | 54.1 ± 14.9 | 51.4 ± 13.1 | 57.4 ± 16.4 | 0.003  6.0 (2.1- 9.9) | NS (0.9) |
| **Female sex** | 212 (74.6%) | 135 (77.1%) | 77 (70.6%) | NS (0.3) | 164 (72.9%) | 92 (73.6%) | 72 (72%) | NS (0.9) | NS (0.7) |
| **Worse admission status (WFNS ≥4)** | 126 (44.4%) | 53 (30.3%) | 73 (67%) | < 0.0001  5 (2.5- 10) | 116 (51.6%) | 39 (31.2%) | 77 (77%) | < 0.0001  10 (3.3- 10) | NS (0.1) |
| **Early hydrocephalus** | 162 (57%) | 83 (47.4%) | 79 (72.5%) | < 0.0001  2.9 (1.7- 4.9) | 147 (65.3%) | 67 (53.6%) | 80 (80%) | < 0.0001  3.5 (1.9- 6.3) | NS (0.08) |
| **Shunt dependence** | 53 (18.7%) | 23 (13.1%) | 30 (27.5%) | 0.002  2.5 (1.4- 4.6) | 47 (20.9%) | 19 (15.2%) | 28 (28%) | 0.02  2.2 (1.1- 4.2) | NS (0.8) |
| **Fisher grade 3** | 202 (71.1%) | 118 (67.4%) | 84 (77.1%) | NS (0.1) | 166 (73.8%) | 83 (66.4%) | 83 (83%) | 0.005  2.5 (1.3-4.7) | NS (0.5) |
| **DCI** | 56 (19.7%) | 21 (12%) | 35 (32.1%) | < 0.0001  3.5 (1.9- 6.4) | 45 (20%) | 19 (15.2%) | 26 (26%) | 0.04  2.0 (1.0- 3.8) | NS (1) |
| **Coil** | 132 (46.5%) | 84 (48%) | 48 (44%) | NS (0.6) | 99 (44%) | 58 (46.4%) | 41 (41%) | NS (0.4) | NS (0.6) |
| **Right-sided infarctions** | 76 (26.8%) | 38 (21.7%) | 38 (34.9%) | 0.01  1.9 (1.1- 3.3) | 7 (3.1%) | 4 (3.2%) | 3 (3%) | NS (1) | < 0.0001  11.4 (5.1-25.2) |
